# The Effects of Temperature and Growth Phase on the Lipidomes of *Sulfolobus islandicus* and *Sulfolobus tokodaii*

**DOI:** 10.3390/life5031539

**Published:** 2015-08-25

**Authors:** Sara Munk Jensen, Vinnie Lund Neesgaard, Sandra Landbo Nedergaard Skjoldbjerg, Martin Brandl, Christer S. Ejsing, Alexander H. Treusch

**Affiliations:** 1 Department of Biology and Nordic Center for Earth Evolution, University of Southern Denmark, Odense 5230, Denmark; E-Mails: saramj@biology.sdu.dk (S.M.J.); vinee11@student.sdu.dk (V.L.N.); saskj11@student.sdu.dk (S.L.N.S.); 2 Department of Physics, Chemistry and Pharmacy, University of Southern Denmark, Odense 5230, Denmark; E-Mail: mmb@sdu.dk; 3 Department of Biochemistry and Molecular Biology, VILLUM Center for Bioanalytical Sciences, University of Southern Denmark, Odense 5230, Denmark; E-Mail: cse@bmb.sdu.dk

**Keywords:** archaea, *Sulfolobus*, *S. islandicus*, *S. tokodaii*, membrane, temperature, growth, tetraether lipid, mass spectrometry, lipidomics

## Abstract

The functionality of the plasma membrane is essential for all organisms. Adaption to high growth temperatures imposes challenges and *Bacteria*, *Eukarya*, and *Archaea* have developed several mechanisms to cope with these. Hyperthermophilic archaea have earlier been shown to synthesize tetraether membrane lipids with an increased number of cyclopentane moieties at higher growth temperatures. Here we used shotgun lipidomics to study this effect as well as the influence of growth phase on the lipidomes of *Sulfolobus islandicus* and *Sulfolobus tokodaii* for the first time. Both species were cultivated at three different temperatures, with samples withdrawn during lag, exponential, and stationary phases. Three abundant tetraether lipid classes and one diether lipid class were monitored. Beside the expected increase in the number of cyclopentane moieties with higher temperature in both archaea, we observed previously unreported changes in the average cyclization of the membrane lipids throughout growth. The average number of cyclopentane moieties showed a significant dip in exponential phase, an observation that might help to resolve the currently debated biosynthesis pathway of tetraether lipids.

## 1. Introduction

All cellular life separates the contents of a cell from the environment using membranes. With these barriers, cells can control and regulate uptake and secretion of compounds and ions, but also energy coupling and conversion take place here. Membranes are mainly composed of lipids and proteins and by regulating the contents and types of those, cells can adapt to the different environmental conditions they are facing. Prokaryotes have been shown to thrive in a wide range of habitats, making it interesting to study how they evolutionarily managed to adapt their membranes and ultimately their cells to all these conditions.

**Figure 1:**
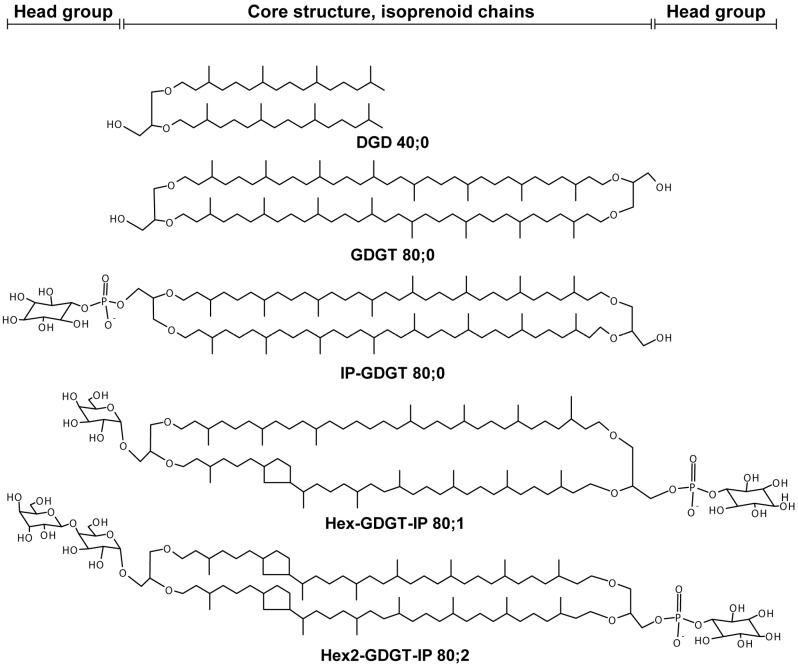
Selected structures of lipids identified in *Sulfolobus islandicus*. DGD 40;0 represent di-glycerol-dialkyl ether with 40 carbons and no double bounds in the isoprenoid chains. GDGT (glycerol-dialkyl-glycerol-tetraether) 80;0 contains zero cyclopentane moieties. IP-, Hex and Hex2 represent inositol, hexose, and dihexyl, respectively.

Especially organisms of the domain *Archaea* can be found in a multitude of environments that are considered “extreme” and it is hypothesized that the ether lipids their membranes consist of enable them to thrive under these conditions [1]. Membranes composed of mainly ether lipids connected to isoprenoid chains are one of the characteristics that distinguish *Archaea* from *Bacteria* and *Eukarya*. Archaeal ether lipids are comprised of glycerol-1-phosphate linked via ether bonds to hydrocarbon chains [2]. Besides these polar diether lipids (DELs), bipolar tetraether lipids (TELs), forming monolayer membranes, have been described in many archaea [3]. The biosynthesis of DELs is now fairly well understood (for a review see [2]), however, how TELs are synthesized is still unclear. One proposed mechanism is the head-to-head condensation of saturated diethers with a later cyclization [2]. Conflicting results from *in vivo* studies [4,5,6] led to the proposal of a new mechanism by Villanueva and colleagues [7]. They suggested a pathway in which the rings are already present in the prenyl chains before they are coupled to the glycerol, requiring enzymes that can accommodate a wider range of substrates. The adaption of archaeal membranes to high temperatures has been the focus of several studies [8,9,10,11,12]. In *Euryarchaeota*, which contain both, DELs and TELs in their membranes [7] a shift of the DEL:TEL ratio towards a higher content of TELs appears to be the mechanism for heat adaption [13]. In *Crenarchaeota*, however, the majority of the membrane lipids are TELs [14], and here the adaption to heat seems to be accomplished by the incorporation of more cyclopentane moieties into the isoprenoid chains [15]. This strategy for coping with high temperatures is well established for hyperthermophilic archaea. It is not well known, however, how the cyclopentane moieties change during the growth of a culture, mainly because only a few studies have investigated the effects of growth phase on the lipid composition [13,16,17,18,19,20]. Organisms in the order *Sulfolobales* within the phylum *Crenarchaeota* serve as model organisms for studies of e.g., gene expression (e.g., [21]), archaeal viruses (e.g., [22]) and population genomics (e.g., [23]) in hyperthermophiles. Not surprising, multiple studies have also analyzed the lipid compositions of *Sulfolobus acidocaldarius*, *S. solfataricus* and *S. shibatae* [8,24,25]. In the frame of these studies, the lipid classes glycerol-dialkyl-glycerol-tetraether (GDGT) and glycerol-dialkyl-nonitol-tetraether (GDNT) have been described, with different head groups such as hexose moieties, inositol phosphate and sulfo groups adding variability to the core lipid structures (Figure 1). In 1994, Zillig and colleagues isolated several *Sulfolobus* species from Icelandic solfataras, which grow optimally at 65 °C–85 °C and pH 2–4 [26]. Subsequently other studies showed that strains of *S. islandicus* were also present in the hot springs of Yellowstone National Park and Lassen Volcanic National Park in the United States [23,27]. While *S. islandicus* has become a model organism for evolutionary genomics [28], its lipid composition has only recently been described [29]. Another archaeal species with an uncharacterized lipidome is *Sulfolobus tokodaii*. It was isolated from Beppu Hot spring in Japan and was initially known as *Sulfolobus* sp. strain 7 [30]. *S. tokodaii* is an obligate aerobe that grows optimally at 75 °C–80 °C and pH 2–3.

High resolution-based shotgun lipidomics has recently been applied for the first time to archaeal species for analyzing TELs [29]. It is a powerful tool that allows the analysis of compounds in a sample without any separation prior to mass spectrometric (MS) analysis [31]. The technique is very sensitive and can analyze low amounts of sample [31,32]. One of the main advantages of the method is the short analysis time. If a complete overview of the lipidome is desired, the time to acquire the data is less than 20 min per sample, compared to up to 1.5 h for LC-MS [33,34]. Challenges with shotgun lipidomics are the identification of isobaric compounds and to distinguish cyclopentane moieties from double bonds as identical masses are detected. However, isobaric compounds can be identified by subsequent fragmentation and the analysis of the fragment patterns [31].

The use of shotgun lipidomics with its low requirements for sample biomass presented a great opportunity to analyze samples taken throughout a growth curve, including those from the low biomass lag phase [35]. A combined study of the effects of temperature and growth phase on the composition of the lipidomes of a crenarchaeal species by analyzing intact polar lipids has, to our knowledge, not been performed previously. Here we present a study of the lipidomes of *Sulfolobus islandicus* and *Sulfolobus tokodaii* at different temperatures and during different growth phases using a state-of-the-art shotgun lipidomics approach.

## 2. Materials and Methods

### 2.1. Chemicals

Casamino acids and yeast extract were purchased from Merck A/S (Hellerup, Denmark). The following chemicals were purchased from Sigma–Aldrich (St. Louis, MO, USA): ammonium acetate, acetic acid, glucose, potassium phosphate, methylamine, ammonium sulfate, magnesium sulfate, ferric chloride, manganese(II) chloride, zink sulfate, copper sulfate, vanadyl sulfate, sodium molybdate, sodium tetraborate decahydrate, calcium chloride, and saccharose. Methanol and Chloroform (both HPLC-grade) were purchased from Rathburn (Walkerburn, Scotland). Main polar lipid (Hex-GDGT-PG) from *Thermoplasma acidophilum* was purchased from Matreya LLC (State College, PA, USA) and internal lipid standard PI 17:0/14:0 and IS PE O-20:0/O-20:0 were purchased from Avanti Polar lipids (Alabaster, AL, USA).

### 2.2. Cell Culture

*S. islandicus* Y.N.15.51 [23] was grown heterotrophically at 70 °C, 75 °C and 80 °C in 150 mL Deutsche Sammlung von Mikroorganismen und Zellkulturen (DSMZ) medium 182 (http://www.dsmz.de/). *S. tokodaii* (strain DSMZ 16993) was grown heterotrophically at 75 °C, 80 °C and 85 °C in 150 mL Brock medium [36] with 0.1% yeast extract and 0.2% saccharose. The cultures were adapted to the actual temperatures in the experiment by a 2 °C rise in temperature over three days prior to the experiment. The adapted cultures were in early stationary phase when they were used as inocula for the cultures of the main experiments. Growth was monitored by optical density at 420 nm. Cells were collected at three time points, during the lag phase (t = 2 h), the exponential phase (t = 20 h) and the stationary phase (t = 70 h) by centrifugation (4000 g, 5 min). After centrifugation, the cell pellet was resuspended in 1.5 mL 155 mM ammonium acetate solution. The collected cell pellet was stored at −80 °C until lipid extraction. As a blank, a culture flask with sterile medium was incubated at 75 °C and samples were taken and processed identical to the cultures. For each temperature, cultures were run in triplicate (biological replicates), except for 75 °C where sampling was performed on duplicates.

### 2.3. Lipid Extraction

The lipids were extracted as described in Jensen *et al*. [29]. In brief, the cell pellet was disrupted by glass bead lysis (15 min, 1400 rpm, Eppendorf Thermomixer comfort). An aliquot of the cell lysate corresponding to 0.4 OD units/mL was transferred to a new tube and diluted with 155 mM ammonium acetate solution to yield a final volume of 200 μL. Because of the limited biomass in the cultures, less cell material (0.2 OD unit/mL) was used for the 2 h time point in comparison to the other time points. One of the technical replicates was spiked with an internal standard mix containing PI 17:0/14:1 (110 pmol) and MPL (750 pmol) at the time of extraction. The others had standards spiked in at the time of the MS analysis. Subsequently, 990 μL chloroform /methanol (2:1, *v*/*v*) were added to each sample followed by vortexing for 30 min at 1400 g and 4 °C. The samples were subsequently centrifuged for 2 min at 1000 g and 4 °C. The lower organic phase was collected and evaporated in a Hetovac evaporator system (Heto, High Technology of Scandinavia, Birkerod, Denmark). The lipid extract was re-dissolved in 100 μL chloroform/methanol (1:2, *v*/*v*). Prior to mass analysis, aliquots of the lipid extracts were diluted 2-fold with 0.01% methylamine in methanol for runs in negative mode. Three extractions were performed from two cell pellets per culture and sampling time point, referred to as technical replicates. Each extraction was injected multiple times at the MS, which we will refer to as analytical replicates.

### 2.4. Structural Analysis by Hybrid Ion-Trap Orbitrap Mass Spectrometry

The analysis of lipids was performed in negative mode using an LTQ Orbitrap XL mass spectrometer (Thermo Fisher Scientific, Bremen, Germany) equipped with a TriVersa NanoMate ion source (Advion Biosciences, Ithaca, NY, USA) [32]. The settings for negative mode were ionization voltage at −0.96 kV and a gas pressure at 0.60 psi. Fourier transform mass spectrometry (FTMS) spectra were recorded in the mass range from 200–2000, 600–900, and 1200–2000. The target mass resolution was set to R = 100,000 (FWHM at *m*/*z* 400).

### 2.5. Data Analysis

The data analysis was performed using ALEX (Analysis of Lipid Experiments) [37]. The target list contained the following tetraether lipid classes: Sulfono-Hex3-GDGT-IP, Hex2-GDGT-IP, Hex-GDGT-IP, IP-GDGT, Hex-GDGT-PG, Sulfo-Hex-GDNT, GDGT, GDNT, Hex-GDNT with 80 carbon atoms in total and from 0 to 8 cyclopentane moieties and diether lipid classes, IP-DGD, PG-DGD, PE-DGD with 40 carbon atoms (Figure 1). These lipid classes were included as they have been described for *S. islandicus* previously and in other *Sulfolobus* species [29,38,39]. As lock mass in the low mass range either PE O-20:0/O-20:0, spiked in just before MS analysis, or PI 17:0/14:1, spiked in before the extraction, were used. For the high mass range Hex-GDGT-PG 80:0 was used as lock mass. A threshold of 2000 counts at the FTMS was applied to filter out noise. Applying the threshold resulted in the exclusion of the lipid classes Hex-GDGT-IP, Sulfo-Hex-GDNT, PG-DGD, and PE-DGD from the analysis because of their low abundance in the samples. After the threshold the measured intensity of each lipid species was corrected for isotopes as described below. The corrected intensities were expressed as percent of total lipid species in all the lipid classes in the sample to correct for variations in the intensities of the technical replicates. This type of approach was chosen over a calculation of mol percent due to the fact that only one internal standard for tetraether lipids is commercially available, which doesn’t cover the full lipid diversity needed here. Thereby the relative abundance of the lipid species is not corrected for their relative response factors. Averaging of values was performed on analytical replicates, on technical triplicates (omitting zero values) and on the biological triplicates, resulting in the final dataset.

### 2.6. Isotope Correction

For the quantification of lipid species in shotgun lipidomics it is important to correct for isotope distribution [31]. This was done for the tetraether lipid classes IP-GDGT, Sulfono-Hex3-GDGT-IP and Hex2-GDGT-IP. The measured intensities are corrected according to Han, 2005, by the following equation [31]: I(corrected) = I(M + 4) − I(M0) × γ(M0) − I(M + 2) × γ(M + 2). The factor (γ), was calculated by the following equation: γ = η (M + 2)/η (M0).

The isotope possibility value (η) was estimated by ALEX (by the software “Extractor”). For each lipid class this factor (γ) was calculated for second and forth isotope and the dataset were corrected for both. All presented values are corrected for isotopes.

### 2.7. Statistical Analysis

To analyze the reduced cyclization of the three different lipid classes Hex2-GDGT-IP, IP-GDGT, and Sulfono-Hex3-GDGT-IP during exponential growth, the relative decrease of the average cyclization number of the replicates between the time points 2 h and 20 h were determined for each growth temperature and archaeal species. This percentage was linearly correlated with the average doubling time of the respective culture.

Non-metric multidimensional scaling (NMDS) analyses were performed in Primer 6 [40] using the targeted lipids of this study (Sulfono-Hex3-GDGT-IP, Hex2-GDGT-IP, IP-GDGT). All analytical replicates above an intensity of 50,000 were included in the analysis. Additionally, outliers showing less than 50% similarity to any other sample in a cluster analysis were removed. Separated by species, the data was transformed to presence/absence and similarities were calculated using the Bray Curtis resemblance measure. The resemblance matrix was plotted with non-metric multidimensional scaling [41] and environmental parameters like sampling time and temperature were overlaid onto the plot using Pearson correlation.

## 3. Results

In order to elucidate the differences in lipid composition at different growth temperatures and follow changes throughout the growth, *S. islandicus* and *S. tokodaii* were cultivated at three different temperatures. Samples were taken during the lag phase (2 h), exponential phase (20 h) and stationary phase (70 h). Lipid extractions were performed in triplicate and the extracts analyzed by high-resolution shotgun lipidomics.

### 3.1. Growth Curves

**Figure A1:**
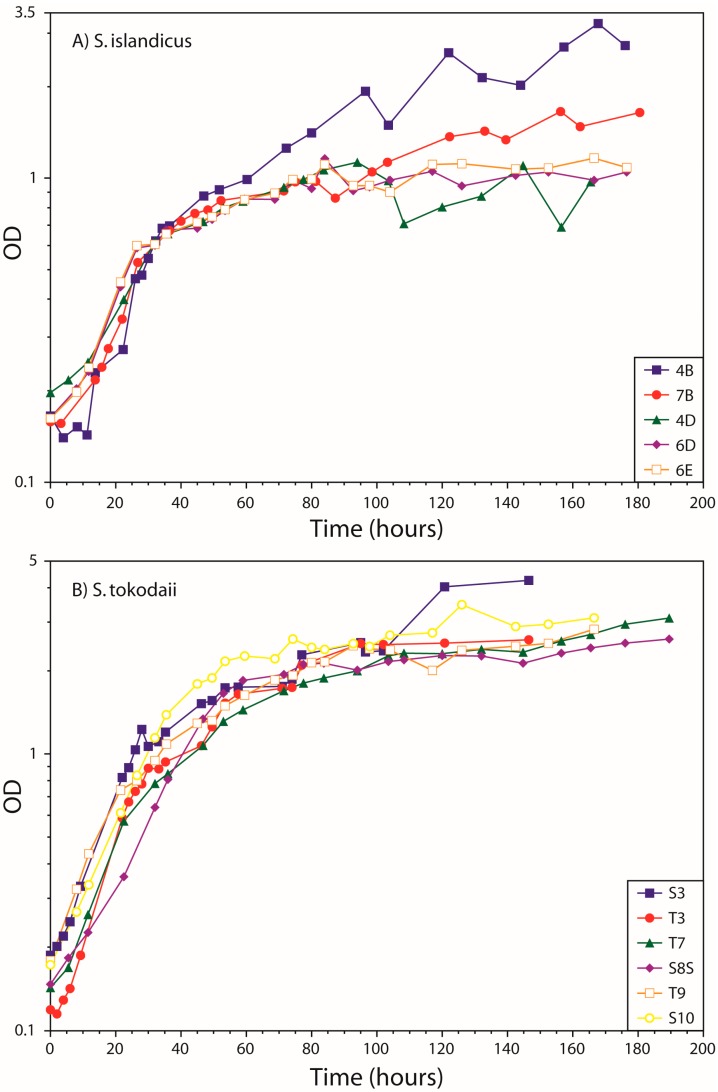
Growth curves of (**A**) *S. islandicus* and (**B**) *S. tokodaii* at 75 °C. A total of 5 (*S. islandicus*) and 6 (*S. tokodaii*) detailed growth curves were recorded in a preliminary experiment to determine the time points for sampling for the main experiment.

In a preliminary experiment detailed growth curves of *S. islandicus* and *S. tokodaii* were recorded at 75 °C (Figure A1) in order to determine the ideal sampling time points for exponential and stationary phase. *S. islandicus* (Figure A1A) showed exponential growth with doubling times of around 11 h up to approx. 30 h of culturing time, followed by a long transition into stationary phase, which started at approx. 60 h with an OD_420_ of around 1. Interestingly, the five parallel cultures showed different behaviors in the stationary phase, some with large fluctuations, which we attribute to evaporation effects of the medium that may have affected the cultures differently. *S. tokodaii* (Figure A1B) showed less fluctuations in stationary phase, which it reached at around 70 h with an average OD_420_ of 3. Doubling times in the exponential phase were approx. 10 h.

**Figure 2:**
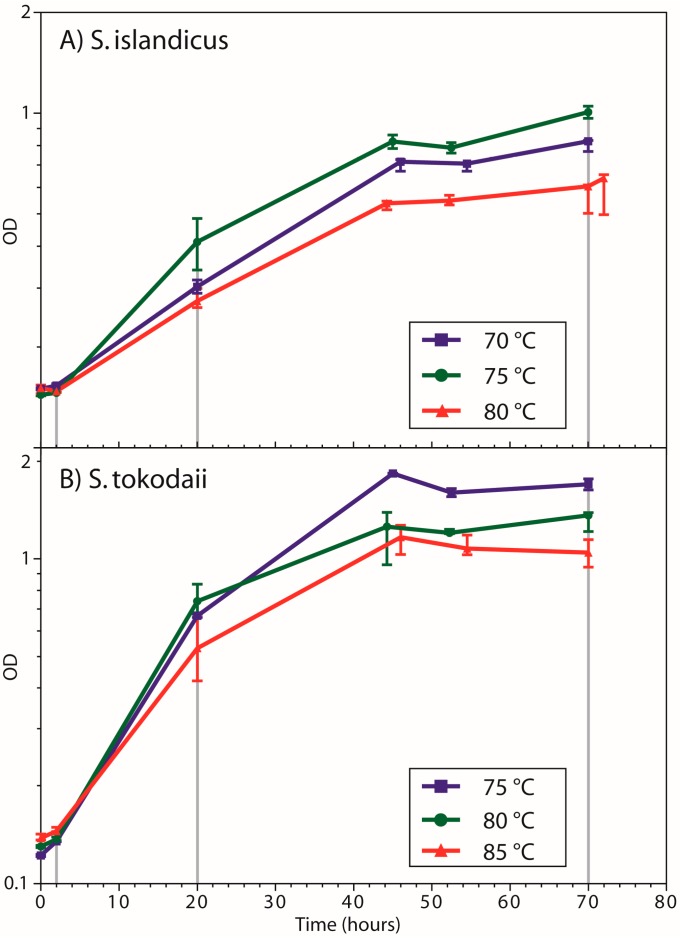
Growth curves of (**A**) *S. islandicus* and (**B**) *S. tokodaii* at the three different temperatures analyzed. For each temperature the median of three replicates is shown and error bars represent the range of values. Sampling times for the analysis of the lipidomes are indicated by grey vertical lines.

For the main experiment *S. islandicus* was cultivated at 70 °C, 75 °C and 80 °C, and *S. tokodaii* was cultivated at 75 °C, 80 °C and 85 °C, respectively. The difference in the temperature ranges studied between the two species is a result of the fact that *S. islandicus* did not show any growth at 85 °C. As predicted from the preliminary experiment, cultures of *S. islandicus* and *S. tokodaii* were at 2 h still in the lag phase at all temperatures (Figure 2). For *S. islandicus* (Figure 2A) the doubling times determined were 19.7, 17.6, and 22.7 h (70 °C, 75 °C and 80 °C, respectively). All cultures reached stationary phase at around 45 h with OD_420_ measurements of 0.82 and 0.64 (70 °C and 80 °C cultures, respectively) after 70 h, which were lower compared to the 75 ° C cultures at an OD_420_ of 1. *S. tokodaii* (Figure 2B) had doubling times of 7.8, 7.4, and 9.6 h (75 °C, 80 °C and 85 °C, respectively). The cultures reached stationary phase at around 45 h and the final OD_420_ measurements determined at 70 h were 1.7, 1.36, and 1.05 (75 °C, 80 °C, and 85 °C, respectively). All cultures were in exponential phase when sampling was performed at 20 h and in stationary phase when sampling at 70 h. 75 °C resulted in the highest yields of biomass for both archaea, however for *S. tokodaii* the 80 °C cultures had the fastest doubling time.

### 3.2. Lipid Composition

**Figure 3:**
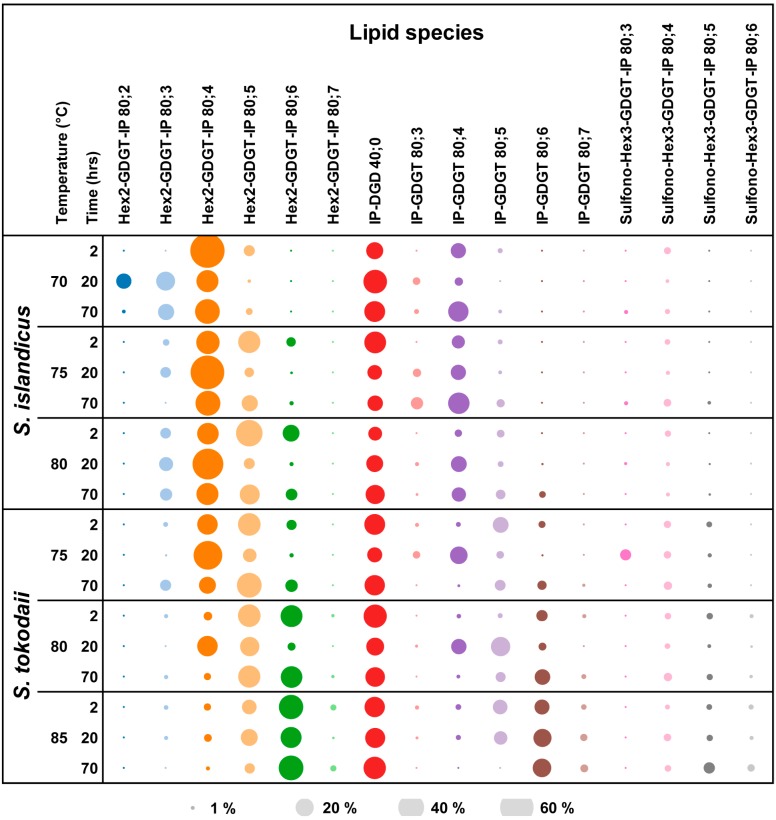
Changes in lipid composition as a function of temperature and growth phases. For both archaea the intensity [%] of the different lipid species are represented by the size of the bubbles. The data the graph is based on are presented in Table 1.

The dataset contained more than 100 peaks per sample after noise filtration with a threshold at 2000 counts for intensity. To focus on the most abundant lipids, the data were analyzed using a targeted approach. Lipid classes we targeted in both archaeal species were Sulfono-Hex3-GDGT-IP, Hex2-GDGT-IP, Hex-GDGT-IP, IP-GDGT, Hex-GDGT-PG, Sulfo-Hex-GDNT, GDGT, GDNT, Hex-GDNT, IP-DGD, PG-DGD, PE-DGD. These lipid classes were included as they have been described for *S. islandicus* and other *Sulfolobus* species previously [29,38,39]. After noise filtration the GDGT, Hex-GDGT-IP and Sulfo-Hex-GDNT, PE-DGD, PG-DGD were excluded from further analyses because of their low abundances. The only commercially available tetraether lipid standard Hex-GDGT-PG, which we used in our study, is a lipid extract from *Thermoplasma acidophilum* and contains contaminating amounts of the lipid classes GDNT and Hex-GDNT. These therefore had to be excluded from the analysis as well. It was not possible to correct the relative abundances of the lipid classes for their response factors, which means that the presented relative amounts of lipid for each lipid species could be different. However, it became clear that the composition of lipids in the two archaea changed both in response to temperature and growth phase (Figure 3).

**Table 1:** Distribution of lipid species.

	Temp	Hours	Lipid class/number of rings in [%]																			
			Hex2-GDGT-IP							IP-GDGT							Sulfono-Hex3-GDGT-IP				IP-DGD	DEL:TEL ratio
			1	2	3	4	5	6	7	1	2	3	4	5	6	7	3	4	5	6	0	
***S. islandicus***	70	2				63	6						12	1				2			15	1:6
		20	1	12	19	26	1			1	3	3	3								30	1:2
		70			14	32	2				1	1	22				1	1			23	1:3
	75	2			2	29	26	4			1		8	1				1			26	1:3
		20			6	61	5					3	12								11	1:8
		70				32	14					9	24	3			1	3			12	1:7
	80	2			6	24	37	15					3	3				2			10	1:9
		20			11	51	6						13	2							15	1:6
		70			7	27	21	7					11	5	2						19	1:4
***S. tokodaii***	75	2			1	22	27	5					1	14	2			2	1		23	1:3
		20				44	10					3	16	3			7	3	1		12	1:7
		70			6	15	33	8						6	4			4	1		21	1:4
	80	2			1	4	27	25					1	1	7	1		2	2	1	29	1:2
		20				22	20	3					12	20	3			1			16	1:5
		70			1	3	27	23						5	12	1		4	2		20	1:4
	85	2			1	3	11	31	2			1	2	11	12	1		1	2	1	22	1:4
		20				3	13	23					1	10	18	3		3	2	1	22	1:4
		70				1	6	32	2						18	3		2	6	3	27	1:3

The values are an average of three technical replicates, three biological replicates. 100% are the total target lipid pool. Standard error (SEM) values are provided in Table A1.

In *S. islandicus* the two main lipid classes observed were Hex2-GDGT-IP and IP-GDGT. At all temperatures Hex2-GDGT-IP was most abundant in the lag and exponential phases (Table 1). Highest amounts of IP-GDGT were detected in the stationary phase, indicating a shift in lipid classes. Additionally, the number of different species in the lipid classes changed over the growth phases as well. In both lipid classes the number of lipid species varied from 2 to 5, with no general trend of more lipid species in certain phases or at certain temperatures (Figure 3). Differences between the two lipid classes showed in the presence of lipid species with different number of cyclopentane moieties. For Hex2-GDGT-IP, lipid species with 3 to 6 cyclopentane rings were observed compared to IP-GDGT, for which lipid species with 4 to 6 rings were detected. The relative abundance of Sulfono-Hex3-GDGT-IP lipids was lower than the other three lipid classes at all temperatures and time points. Diether lipids were detected in our dataset as well, with the lowest amount in the lag phase at the highest temperature and the highest amount in the exponential phase at 70 °C. The ratio of diether:tetraether lipids was highest in the lag phases at 70 °C and 80 °C (1:6 and 1:9, respectively) and highest in the exponential phase at 75 °C (1:8).

*S. tokodaii* showed differences in the lipid composition compared to *S. islandicus*, most notably in the higher abundance of Sulfono-Hex3-GDGT-IP. The total amount of Hex2-GDGT-IP was highest in the lag phase, except at the lowest temperature. For IP-GDGT the highest amount was detected in the exponential phase, which is in contrast to *S. islandicus*. The distribution of lipid species within the lipid classes in *S. tokodaii* was similar for both, Hex2-GDGT-IP and IP-GDGT, with four to six rings in each. Further, a shift towards more cyclopentane moieties at higher temperatures could be observed in all lipid classes. The amount of diether lipid was highest in the lag phase at 75 °C and 80 °C and in stationary phase at 85 °C. The ratio of diether:tetraether lipids was highest in the exponential phase at all temperatures (1:7, 1:5 and 1:4, 75 °C, 80 °C and 85 °C, respectively).

### 3.3. Ring Distribution at Different Temperatures

**Figure 4:**
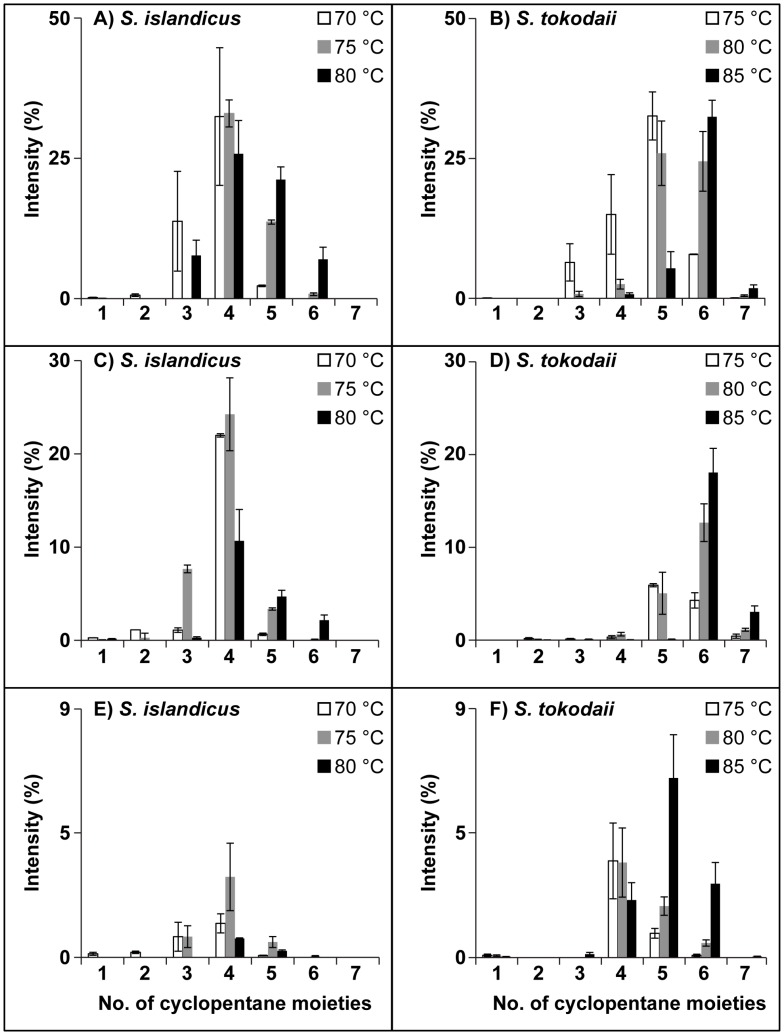
Overview over changes of lipid species in the different lipid classes with increasing growth temperature. The relative intensity of the lipid species in stationary phase (70 h) and averages of three biologics replicates are shown. (**A** + **B**) Hex2-GDGT-IP, (**C** + **D**) IP-GDGT, (**E** + **F**) Sulfono-Hex3-GDGT.

**Figure A2:**
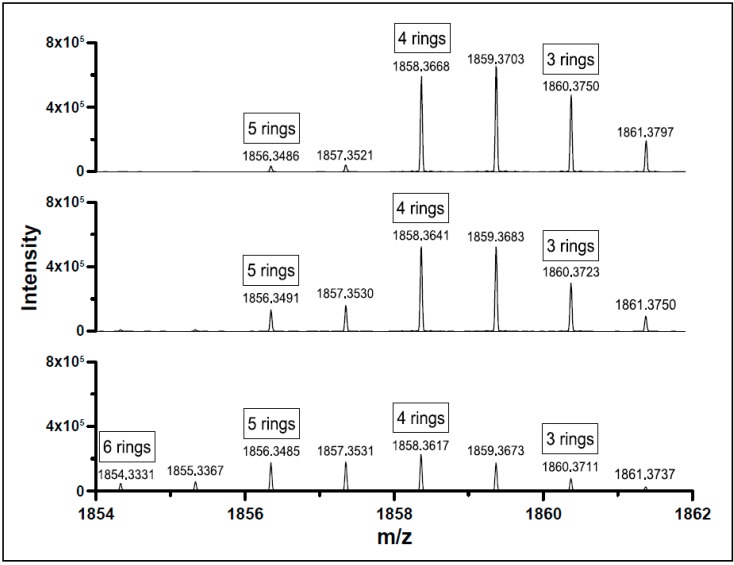
Increasing number of cyclopentane moieties in the lipid class Hex2-GDGT-IP with higher temperatures. A selection of three FTMS spectra of from *S. islandicus* are shown here. (**A**) 70 °C; (**B**) 75 °C; (**C**) 80 °C; at stationary phase (70 h). The peaks for the lipid species with 3 to 5 rings can also contain for isotope peask of the lighter lipid species.

To explore the trend that cultures grown at higher temperatures contained more cyclopentane moieties in the core structures of their lipids, we focused on the 70 h time point for all three temperatures. It already became clear when zooming in on the most abundant lipid class, Hex2-GDGT-IP, directly in the FTMS spectra, that more cyclopentane moieties appeared with higher growth temperature. The spectra showed a shift in the mass peak distribution along with changes in the temperature (Figure A2). At 70 °C the abundant peak in the cluster was at *m/z* 1858.3668, corresponding to the lipid species Hex2-GDGT-IP with four cyclopentane moieties and a minor amount of both three and five cyclopentane moieties (*m*/*z* 1860.3750 and *m*/*z* 1856.3486, respectively). Both, increasing the temperature by 5 and 10 degrees increased the amount of lipids with five and six cyclopentane moieties and decreased the amount of those with three cyclopentane moieties. This trend could be observed in all biological replicates. For *S. islandicus* grown at 70 °C most of the lipid species observed had four cyclopentane moieties, whereas at 80 °C the abundant lipid species had either four or five (Figure 4). Additional lipid species with six cyclopentane moieties were detected at this temperature as well. Similarly to Hex2-GDGT-IP, the most common lipid species in IP-GDGT had four rings at the lowest temperature and five and six rings at higher temperatures. The third lipid class detected in *S. islandicus*, although at low abundances, was Sulfono-Hex3-GDGT-IP, which had at 70 °C lipid species with three and four rings. With an increase in temperature more lipid species with five and six rings appeared.

In *S. tokodaii* the shift in lipid species towards those with more cyclopentane moieties with increased temperature was even more pronounced. For Hex2-GDGT-IP the major lipid species contained five cyclopentane moieties at 75 °C, whereas at 85 °C the lipid species with six cyclopentane moieties was most abundant. The lipid distribution for IP-GDGT was similar but not as pronounced. For Sulfono-Hex3-GDGT-IP the abundant lipid species had four rings at 75 °C and shifted to five and six rings with an increase in temperature. The comparison of the two archaeal species showed a difference in the number of cyclopentane moieties in the core structure of the lipids. For Hex2-GDGT-IP and IP-GDGT, lipids with four cyclopentane moieties were the most abundant ones in *S. islandicus*, whereas in *S. tokodaii* it were the lipid species with five and six cyclopentane moieties. In *S. islandicus* the abundant lipid species of Sulfono-Hex3-GDGT-IP had, similarly to the other two lipid classes, four cyclopentane moieties, however in *S. tokodaii* lipid species with both four and five rings were observed.

**Figure 5:**
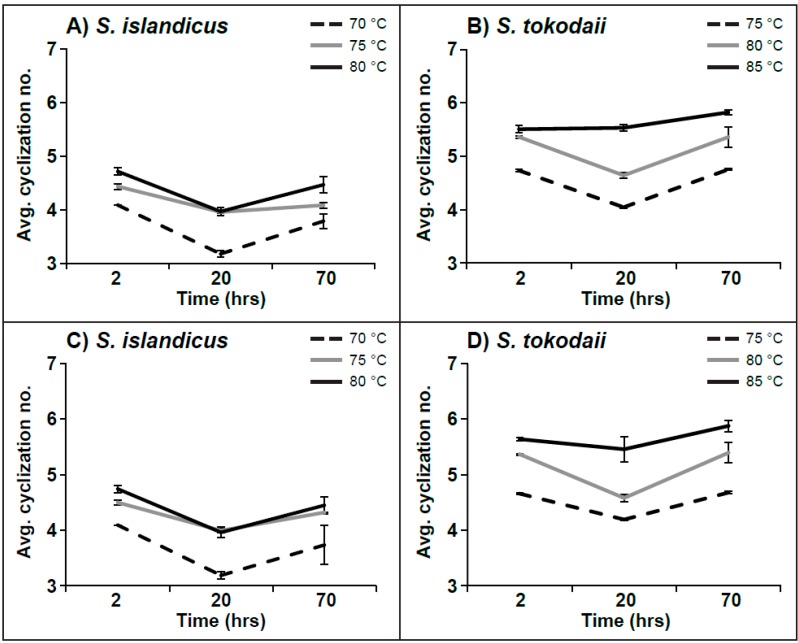
Dip of the average number of cyclopentane moieties (avg. cyclization) in the total lipid pool (**A**) and (**B**) and lipid class Hex2-GDGT-IP (**C**) and (**D**) in exponential phase, observed in (**A**), (**C**) *S. islandicus*, (**B**), (**D**) *S. tokodaii* at all three temperatures. Values for IP-GDGT and Sulfono-Hex3-GDGT-IP are presented in Table 2. *n* = 3.

In order to better follow how the number of cyclopentane moieties were changing over different parameters, an average cyclization number was calculated [8]. In this analysis we investigated both time and temperature, which gave the opportunity to follow how the number of cyclopentane moieties changed over these parameters. For the bulk lipid the average number of cyclization showed a decrease from lag phase to exponential phase for all three temperatures for *S. islandicus* and at for *S. tokodaii* at 75 °C and 80 °C. This brief decrease was followed by an increase to the stationary phase. The changes were most pronounced at the lowest temperatures in each archaeon (70 °C or 75 °C, respectively) (Figure 5). The same picture appeared for both archaea and all three lipid classes (Table 2), but was less pronounced for Sulfono-Hex3-GDGT-IP. We suspected the reason behind the observation in a lacking speed of lipid cyclization during biosynthesis at high growth rates. Therefore, we correlated the doubling times of the different cultures with the respective relative decrease in average cyclization. For *S. tokodaii* the correlations were strong (−0.97, −0.98, 0.99 for Hex2-GDGT-IP, IP-GDGT, Sulfono-Hex3-GDGT-IP, respectively) in contrast to *S. islandicus* where they were not as pronounced (0.43, 0.21, 0.15 for Hex2-GDGT-IP, IP-GDGT, Sulfono-Hex3-GDGT-IP, respectively).

**Table 2:** Distribution of average cyclization number.

		*S. islandicus*									*S. tokodaii*								
		70 °C			75 °C			80 °C			75 °C			80 °C			85 °C		
Time [h]		Avg.		SD	Avg.		SD	Avg.		SD	Avg.		SD	Avg.		SD	Avg.		SD
**Hex2-GDGT-IP**	2	4.09	±	0.002	4.50	±	0.043	4.74	±	0.067	4.66	±	0.014	5.37	±	0.013	5.64	±	0.026
	20	3.18	±	0.068	3.99	±	0.031	3.96	±	0.099	4.19	±	0.010	4.58	±	0.066	5.46	±	0.223
	70	3.73	±	0.349	4.32	±	0.003	4.45	±	0.156	4.68	±	0.022	5.39	±	0.181	5.88	±	0.105
**IP-GDGT**	2	4.09	±	0.041	3.88	±	0.115	4.53	±	2.133	5.00	±	0.032	5.76	±	0.033	5.43	±	0.159
	20	2.73	±	0.010	3.80	±	0.057	4.03	±	0.030	3.82	±	0.074	4.65	±	0.071	5.59	±	0.133
	70	3.82	±	0.083	3.85	±	0.102	4.46	±	0.209	5.25	±	0.098	5.68	±	0.147	6.05	±	0.031
**Sulfono-Hex3-GDGT-IP**	2	4.00	±	0.000	4.00	±	0.000	4.00	±	1.886	4.38	±	0.143	4.73	±	0.093	5.03	±	0.063
	20	3.42	±	0.051	4.13	±	0.054	3.84	±	0.118	3.46	±	0.232	4.49	±	0.132	4.59	±	0.076
	70	3.39	±	0.176	3.97	±	0.080	4.26	±	0.037	4.17	±	0.043	4.46	±	0.133	5.02	±	0.016

Average number of cyclopentane moieties in the core lipid structure. Calculated as (%monocyclic + 2 × %bicyclic + 3 × %tricyclic + 4 × %tetracyclic, *etc.* /100). A number equal with 4 means all the lipid species in the sample for that temperature and that time points have only four cyclopentane moieties. *n* = 3.

### 3.4. Lipid Composition at Different Growth Phases

As explored earlier, a shift in the lipid composition was detected during growth. At 70 °C the lipid composition of *S. islandicus* changed from a domination of Hex2-GDGT-IP 80;4 (63%) (Table 1) in the lag phase to a more diverse lipid composition with Hex2-GDGT-IP 80;1-5 rings, IP-GDGT 1-4 rings and IP-DGD 40:0 in exponential phase. In stationary phase Hex2-GDGT-IP, IP-DGD and IP-GDGT were relatively evenly distributed. At 75 °C the lipid pool was divided between Hex2-GDGT-IP 80;4 and with five rings and IP-DGD 40;0 (29%, 26% and 26%, respectively) in lag phase, shifting to a domination of Hex2-GDGT-IP 80;4 (61%) in exponential phase and an evenly distribution of lipid classes similar to the 70 °C culture during stationary phase. At 80 °C the lipid pool was mainly Hex2-GDGT-IP 80;3 and four rings (24% and 37%, respectively) in lag phase, changing to being half of the lipids Hex2-GDGT-IP 80;4 (51%) in exponential phase and again to an evenly distribution of abundances between Hex2-GDGT-IP, IP-DGD and IP-GDGT in stationary phase. At all temperatures Sulfono-Hex3-GDGT-IP was mainly detected during lag phase in *S. islandicus*. The amount of IP-GDGT increased from lag phase to stationary phase, with a decrease in the amount of Hex2-GDGT-IP.

The lipid pools of *S. tokodaii* during the lag phases at all three temperatures were comprised of Hex2-GDGT-IP, IP-DGD and IP-GDGT (e.g., at 75 °C Hex2-GDGT-IP with four rings 22% and five rings 27%, IP-DGD 23% and IP-GDGT 80;5 14%). In the exponential phase the cultures grown at 75 °C were dominated by Hex2-GDGT-IP 80;4. For the other two temperatures the lipid distribution was more even between the lipid classes (at 80 °C Hex2-GDGT-IP 80;4 and 80;5 22% and 20%, IP-GDGT 80;4 12% and 80;5 20%, IP-DGD 15%) (Table 1). In the stationary phase the broadest distribution of lipid species was observed at 75 °C, whereas at the other two temperatures Hex2-GDGT-IP 80;6 was more dominant. The highest amounts of Sulfono-Hex3-GDGT-IP were detected in the exponential phase at the lowest temperature and in the stationary phase at the highest temperature. Interestingly, the amount of IP-GDGT decreased from the lag to the stationary phase at the lowest temperature but increased at the other two temperatures. Abundances of Hex2-GDGT-IP were relatively similar throughout the growth phases.

### 3.5. Statistical Analysis of the Dataset

**Figure 6:**
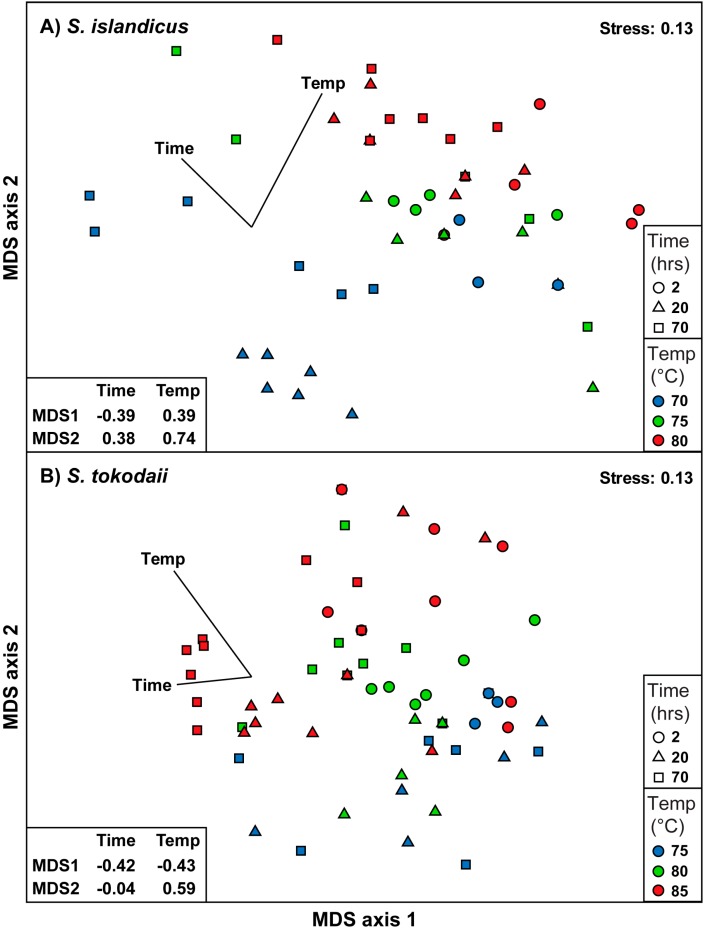
Clustering of samples according to temperature and growth phase. NMDS analysis of the presence/absence transformed dataset of the targeted lipids Hex2-GDGT-IP, IP-GDGT, and Sulfono-Hex3-GDGT-IP using the Bray Curtis resemblance measurement. The Pearson correlations with temperature and time of sampling are shown as vectors. Samples are coded based on time of sampling (shape of symbol) and temperature (color; see legend).

We analyzed our dataset using NMDS statistics. For both *S. islandicus* and *S. tokodaii* the two-dimensional solutions had a stress of 0.13, indicating stable solutions (Figure 6). Overlays with environmental parameters showed significant correlations for both, temperature and time. The strongest trend was observed for temperature, with correlations of 0.39/0.74 (MDS axis 1/axis 2) in *S. islandicus* and −0.43/0.59 in *S. tokodaii* (Figure 6). Less strong but still significant were the correlations for sampling time during the growth curve with −0.39/0.38 and −0.42/−0.04 (MDS axis 1/axis 2) for *S. islandicus* and *S. tokodaii*, respectively.

## 4. Discussion

### 4.1. Growth Curves

From our growth curves we could determine that we indeed sampled lag, exponential, and stationary phases of our cultures (Figure 2). Interesting to note are the subtle deviations between the growth curves of the main experiment and those from the preliminary experiment. While in *S. islandicus* the OD in the stationary phase reached 1 in both experiments, the doubling time in the main experiment was significantly longer (17.6 *vs.* 11 h at 75 °C). In contrast, *S. tokodaii* grew quicker than expected in the main experiment (doubling time 7.4 *vs.* 10 h at 75 °C), however doubled around one time less before reaching stationary phase. These differences can be explained by slightly different culturing conditions between the preliminary and main experiment, notably a higher culture volume and aeration by shaking in the preliminary in contrast to stirring in the main experiment. Under our culturing conditions the optimal growth temperature for *S. islandicus* was estimated to be 75 °C, where the highest cell density was reached. To our knowledge this is the first paper describing cultivation of *S. islandicus* at different temperatures. *S. islandicus* is primarily used as a model for genome evolution and virus experiments, and therefore only few publications have focused on its growth [28]. The local environment where *S. islandicus* strain Y.N. 15.51 was originally isolated from has a temperature of 59 °C [23]. This temperature is significantly lower than the optimal growth temperature estimated under our culturing conditions. The other studied archaeon *S. tokodaii* has a reported optimal growth temperature between 75–80 °C under chemoheterotrophic conditions [30]. This matched our result well.

### 4.2 Lipid Profile

Previous studies have characterized the lipid compositions of *S. acidocaldarius*, *S. solfataricus* and *S. shibatae*, describing the following lipid classes in these organisms: GDGT, GDNT, Hex2-GDGT or Hex-GDNT, IP-GDGT, Hex-GDGT-IP, Sulfo-Hex-GDNT, Hex2-GDGT-IP or Hex-GDNT-IP, IP-DGD, PG-DGD, PE-DGD [8,24,25]. Out of these we could detect IP-DGD, IP-GDGT, Hex2-GDGT-IP and Sulfono-Hex2-GDTG-IP in *S. islandicus* and *S. tokodaii*. The lipid classes Hex-GDGT-IP, Sulfo-Hex-GDNT, GDGT, PE-DGD, and PG-DGD were also detected in some of the samples, but were excluded from the analysis due to their sporadic appearance just around noise level.

To the best of our knowledge, in none of the *Sulfolobus* species changes in lipid composition during growth have been investigated so far. However, other studies have investigated the ratio of diether: tetraether lipids during growth phases in other archaeal species [13,16,17,18,19,20]. Contradictory results came out of these studies: an increase in tetraether lipids toward stationary phase [13,18], the opposite with higher amounts of diether lipids in stationary phase [19], and no pronounced changes in the ratio of diether: tetraether lipids [16,17]. Our results for the analyzed tetraether lipid classes in both archaea showed no clear picture for either increasing or decreasing in the ratio of diether: tetraether lipids. The order *Sulfolobales* belongs to the *Crenarchaeota*, which have mainly tetraether lipids in their membranes [14]. The suggested mechanism to adapt to higher temperatures is the incorporation of more cyclopentane rings into the core structure of the lipids [15]. Perhaps changing the ratio of diether:tetraether lipids plays no or only a minor role in the heat adaption in these organisms. In *Thermoplasma acidophilum* another mechanism to cope with higher temperatures has been observed. Here the head groups changed, with more sugar moieties appearing at higher growth temperatures [10]. While the detected lipid classes in our study contained 3 hexyl, 2 hexyl, and no hexose moieties, no clear trend towards more hexose moieties at higher growth temperatures were observed, mainly because Hex2-GDGT-IP was present at all temperatures (Figure 3).

### 4.3. Ring Distribution at Different Temperatures

Several studies have demonstrated that hyperthermophilic archaea respond to the change in growth temperature by increasing the number of cyclopentane moieties in their membrane lipids [8,9,10,11,12,42,43,44]. In this study we used a shotgun lipidomics approach to investigate if this is also the case in *S. islandicus* and *S. tokodaii*. The results showed that indeed more cyclopentane moieties in the lipid cores appeared with higher growth temperatures (Figure 4). When comparing the two archaeal species at the same temperature, we also observed a difference in the number of cyclopentane moieties in their membrane lipids. Generally, *S. tokodaii* contained one to two cyclopentane moieties more than *S. islandicus* at the same temperature. Comparing this with data available for *S. solfataricus* (2 cyclopentane moieties [8]) and *Sulfolobus sp.* (four cyclopentane moieties [12]) showed that *S. tokodaii* has a higher amount of lipids with a high number of cyclization (six cyclopentane moieties) at the same temperatures. A reason for this finding could be that the environmental temperatures these archaea live in are different, as discussed earlier, and thereby the organisms prefer to synthesize core lipids with a different number of cyclopentane moieties as starting points. From there on they adapt to increasing temperatures by increasing the number of cyclopentane moieties in their lipids. This could be a plausible explanation, however the two species *S. solfataricus* and *S. islandicus* are closely related and therefore their responses at the same temperature should be similar. Another hypothesis to explain the observations made could be an influence of pH, which was different in the cultures from *S. islandicus* (starting pH 4) and *S. tokodaii* (starting pH 3). We didn’t measure the pH in our main experiment as preliminary results have only shown changes in pH of up to 0.5. However, in the literature both a decrease [10] and an increase [42] in cyclization with decreasing pH have been reported. With only our current dataset and the information available in the literature it is impossible to resolve the influence of pH on the observed differences in cyclization between the two species, showing the need for further studies [30].

Including the results from this study, in total 12 archaeal species have been investigated in terms of change in number of cyclopentane moieties with growth temperature [44]. In all studies more cyclopentane moieties were detected in the core structures of the membrane lipids at higher temperatures, except for Yang and Haug, 1979 [8,9,11,12,42,43]. Explanations for this deviation observed by Yang and Haug could be differences in extraction conditions or the pH of the medium as discussed by other studies of the same archaeal species which could show more rings at higher growth temperatures [45].

There are several explanations for the biological and biophysical reasons for increasing the number of cyclopentane moieties of the membrane lipids with temperature [44]. According to Uda *et al*. [43], the thermoacidophilic archaea introduce these cyclopentane rings to maintain a stable fluidity of the membrane against the environmental temperature changes. To increase the stability of the membrane, the lipids need to be packed more tightly, which is achieved by introducing more cyclopentane rings. A previous study using molecular modeling has shown that more rings in the core structure of lipids increases the tightness of membranes [15]. If the reason for having cyclopentane moieties is only the maintenance of membrane functionality at high temperatures, then cyclopentane moieties should only be present in membrane lipids of hyperthermophilic archaea. However, they have also been reported in membranes of mesophilic *Thaumarchaeota*. These organisms can contain both GDGT with cyclopentane moieties and their characteristic core lipid crenarchaeol, with four cyclopentane moieties and one cyclohexyl moiety [46]. These observations indicate that the stabilization of the membrane at higher temperatures is not the only function the cyclopentane moieties serve [47].

### 4.4. Average Number of Cyclization

The average number of cyclization was calculated for each lipid class at the different temperatures as a function of time. For all the temperatures a dip in the average number of cyclization was observed in the exponential phase for each of the lipid classes. This finding has, to our knowledge, not been described before. The same picture appeared for *S. islandicus* at all temperatures and for *S. tokodaii* at 75 °C and 80 °C when the bulk cyclization number was calculated. Only for the *S. tokodaii* cultures at 85 °C a higher average cyclization number was observed in stationary phase.

Since a dip in the average cyclization number appeared both for the total lipid pool and also when analyzing each lipid class separately it can be ruled out that the changes in the average cyclization number are due to changes in the relative amounts of the different lipid classes. In the literature the average number of cyclization is generally calculated for the stationary phase, where the biomass is highest. A comparison of the average numbers of cyclization for the lipid class Hex2-GDGT-IP (or Hex-GDNT-IP) in the order *Sulfolobales* grown at 75 °C revealed that *S. solfataricus* lipids contained the lowest amount of rings (1.74) [8] and *S. tokodaii* the highest (4.68). The two closely related species *S. islandicus* and *Sulfolobus sp.* had average cyclization numbers of 4.32 and 3.93 [12], respectively. This comparison indicates that other factors than temperature are affecting the cyclization of membrane lipids. Some factors that are discussed are e.g., salinity, pressure, nutrients, and pH [44]. However, the influence of pH is not clear, as different trends have been observed. Boyd and colleagues demonstrated an increase of the number cyclopentane moieties with decreasing pH in *Acidilobus sulfurireducens* [42], while Shimada and colleagues showed a decrease with decreasing pH in *T. acidophilium* [10]. These contradicting results indicate a potentially species and/or phylum specific mechanism to cope with changes in pH. However, in the order *Sulfolobales* one could assume that the strains respond to changes in pH with the same mechanisms by either an increase or a decrease in the number of cyclopentane moieties. A reason for the lower average cyclization number for *S. solfataricus* and *Sulfolobus sp.* could be related to pH or unknown factors.

The effects of growth phase on the lipid composition have been investigated in multiple archaea, mainly with a focus on diether to tetraether lipid composition with contradictory results [13,16,17,18,19,20]. However, only the paper from Elling and colleagues is describing the effect that the growth phase has on the number of cyclopentane moieties [18]. They observed a minor decrease in the total GDGT in the late exponential to early stationary phase. It remains to be determined if these observations in species of the phylum *Thaumarchaeota* can be traced back to the same mechanisms as our observations in the crenarchaeal *Sulfolobales* species.

As the observed dip in cyclization gives potential indications on the TEL biosynthesis pathway, we wanted to make sure that the observation is not an artifact. The average number of cyclization could be influenced by the number of lipid species detected in the exponential phase in comparison to the two other phases. However, there was no correlation between the number of lipid species in the sample and the average number of cyclization, which means that the result was not affected by the method.

One could think of several possible explanations for the observed decrease in average cyclization number. First, an actively regulated change in the lipid profile for an unknown biological reason during different growth phases that has not been reported in hyperthermophilic archaea yet. While in the exponential phase, the de-novo synthesis produces lipids with fewer cyclopentane moieties. This needs to be changed during the culture’s transition into the stationary phase, as the membrane of the cell needs to be optimized by introducing lipids with more rings. This hypothesis is based on the assumption that the lipid regulation in *Archaea* is different during different growth phases, but with the limited data so far it is too early to conclude if this is a plausible explanation for our observation. However, these changes in lipid profiles are common in eukaryotes and bacteria [48,49]. The second explanation could be that the division of the cells is too quick, so that the enzymes responsible for the cyclization of the core lipids cannot keep up. This would result in TELs with fewer rings. When the growth slows down towards stationary phase the lipids with less cyclopentane moieties get either further cyclicized by an unknown enzyme or replaced with new lipids that have a higher number of rings. The strong negative correlation of the two main membrane lipids Hex2-GDGT-IP and IP-GDGT with doubling time (−0.97, −0.98, respectively) in *S. tokodaii* supports this theory. However, in *S. islandicus* a weak positive correlation was observed, indicating either unresolved differences between the two archaea or a different explanation of the phenomenon. Our observation fits well with the initially proposed biosynthesis pathway for TELs (summarized in [2]), a head-to-head condensation of saturated diethers with a later cyclization. This could explain the increase in cyclopentane moieties when the cells approach the stationary phase. However, this model would require an enzyme that is active in the membrane to execute the cyclization and can chemically synthesize cyclopentane moieties from saturated carbon chains. The recently proposed alternative biosynthesis pathway of TELs [7], in which the cyclization of tetraether lipids takes place during biosynthesis and not after the condensation of two diethers, would require a significant lipid turnover to match our observations. The removal of less cyclicized lipid species and the replacement with those with more rings would require a significant biosynthetic effort, which, from an ecological point of view, would put species that do this at a disadvantage. Unfortunately no lipid turnover rates are known for hyperthermophilic archaea, making this speculative. Further studies with more samples taken throughout the growth curve are needed to elucidate the mechanism behind the observed result and shed light on the biosynthetic pathway of TELs in archaea.

## 5. Conclusions

In our study, we investigated the effects of temperature and growth phase on the lipidomes of *S. islandicus* and *S. tokodaii* using shotgun lipidomics for the first time. Similarly to other archaea, adaption to higher growth temperatures was achieved by an increase in the number of cyclopentane moieties in the membrane lipids of both, *S. islandicus* and *S. tokodaii*. However, for the same lipid class Hex2-GDGT-IP at the same growth temperature, different average cyclization numbers were observed in the two archaea, which indicates that other factors influence the number of cyclopentane moieties as well. Following the average number of cyclopentane moieties over growth, a dip was observed in the exponential phase at all temperatures. This previously unreported phenomenon hints to the potential biosynthesis pathway of tetraether lipids as it fit well with the traditionally proposed pathway [2], only missing an identified enzyme that can introduce cyclopentane moieties into tetraether lipids in the membrane. In contrast, the recently proposed pathway [7] would require a high tetraether lipid turnover rate in order to fit to our observation. For this reason further studies are needed. It would be especially important to get samples at more time points during all the growth phases in order to better document transitions in lipid composition between the data points shown in this study. Although technically challenging at the moment, the targeted tracing of lipids over their lifespan, from biosynthesis to degradation, would allow the elucidation of their biosynthesis and potential modification in the membrane as well as the determination of membrane lipid turnover rates in hyperthermophilic archaea.

## Appendix

**Table A1:** Relative distribution of lipid species. The values in () are SEM of replicates.

Lipid class	Temp		Hours	Number of rings (SEM values)							
				1	2	3	4	5	6	7	1 *
**Hex2-GDGT-IP**	***S. islandicus***	70	2				63 (0.4)	6(0.1)			15 (1.1)
			20	1 (0.1)	12 (2.4)	19 (2.0)	26 (0.7)	1 (0.1)			30 (1.6)
			70			14 (8.9)	32 (12.3)	2 (0.1)			23 (1.5)
		75	2			2 (1.0)	29 (2.6)	26 (0.5)	4 (0.8)		26 (10.7)
			20			6 (1.4)	61 (2.0)	5 (0.7)			11 (0.7)
			70				32 (2.4)	14 (0.4)			12 (0.4)
		80	2			6 (2.0)	24 (0.8)	37 (1.2)	15 (0.7)		10 (0.9)
			20			11 (3.9)	51 (2.1)	6 (0.8)			15 (1.0)
			70			7 (2.7)	27 (5.9)	21 (2.3)	7 (0.1)		19 (2.2)
	***S. tokodaii***	75	2			1 (0.5)	22 (4.2)	27 (2.8)	5 (0.2)		23 (0.8)
			20				44 (0.2)	10 (0.4)			12 (1.7)
			70			6 (3.3)	15 (7.1)	33 (4.3)	8 (0.1)		21 (10.5)
		80	2			1 (0.3)	4 (0.6)	27 (0.2)	25 (1.4)		29 (1.8)
			20				22 (1.6)	20 (1.9)	3 (0.5)		16 (3.3)
			70			1 (0.5)	3 (0.9)	27 (5.8)	23 (2.3)		20 (2.9)
		85	2			1 (0.3)	3 (0.6)	11 (1.6)	31 (3.9)	2 (0.7)	22 (1.4)
			20				3 (1.8)	13 (5.9)	23 (2.7)		22 (2.3)
			70				1 (0.3)	6 (3.0)	32 (3.0)	2 (0.6)	27 (7.0)
**IP-GDGT**	***S. islandicus***	70	2				12 (1.3)	1 (0.4)			
			20	1 (0.3)	3 (0.4)	3 (0.5)	3 (0.5)				
			70		1 (0.5)	1 (0.4)	22 (3.9)				
		75	2		1 (0.2)		8 (5.7)	1 (0.1)			
			20			3 (0.7)	12 (1.7)				
			70			9 (4.0)	24 (2.4)	3 (0.1)			
		80	2				3 (0.9)	3 (1.0)			
			20				13 (0.3)	2 (0.1)			
			70				11 (3.4)	5 (0.7)	2 (0.6)		
	***S. tokodaii***	75	2				1 (0.1)	14 (8.4)	2 (0.1)		
			20			3 (1.5)	16 (1.9)	3 (0.1)			
			70					6 (0.2)	4 (0.8)		
		80	2				1 (0.1)	1 (0.4)	7 (6.5)	1 (0.3)	
			20				12 (1.8)	20 (2.9)	3 (0.8)		
			70					5 (2.3)	12 (2.0)	1 (0.2)	
		85	2			1 (0.3)	2 (0.4)	11 (3.9)	12 (1.0)	1 (0.2)	
			20				1 (0.6)	10 (3.2)	18 (1.6)	3 (0.7)	
			70						18 (2.6)	3 (0.6)	
**Sulfono-Hex3-GDGT-IP**	***S. islandicus***	70	*2*				2 (0.1)				
			*20*								
			*70*			1 (0.5)	1 (0.3)				
		75	*2*				1 (0.1)				
			*20*								
			*70*			1 (0.4)	3 (1.2)				
**Sulfono-Hex3-GDGT-IP**	***S. islandicus***	80	*2*				2 (0.6)				
			*20*								
			*70*								
	***S. tokodaii***	75	*2*				2 (1.3)				
			*20*			7 (3.3)	3 (1.8)				
			*70*				4 (1.4)				
		80	*2*				2 (0.3)		1 (0.2)		
			*20*				1 (0.3)				
			*70*				4 (1.2)				
		85	*2*				1 (0.1)		1 (0.1)		
			*20*				3 (1.2)		1 (0.3)		
			*70*				2 (0.6)		3 (08)		

* The diether IP-DGD 40;0.
